# A Patient's Protest

**Published:** 1918-12-07

**Authors:** A. M. Dale

**Affiliations:** 8 Oxford Street, Marlborough, Wilts.


*To the Editor of* The Hospital.

### A Patient's Protest.

*To the Editor of* The Hospital.

Sir,—We hear much about altering nurses' hours, but why not start with the patients? There would not be nearly so many night nurses required if patients were started at 6.30 a.m. or even 7 a.m. instead of being washed at 4 a.m. The system is a cruel one, and as a patient I often longed for a whole night's sleep Doctors and night sisters walked and talked in the wards until nearly midnight, and work was always begun by 4 a.m. Then why serve patients with inferior food? At least one large London hospital provides third-rate meat, margarine, and eggs. Quite a different article was bought for the nurses and doctors. The monotony for long cases may mean easy housekeeping, but it nauseates patients to face unending rice pudding.—Yours obediently, (Nurse) A. M. Dale.

8 Oxford Street, Marlborough, Wilts.

